# High-Resolution Recombination Patterns in a Region of Human Chromosome 21 Measured by Sperm Typing

**DOI:** 10.1371/journal.pgen.0020070

**Published:** 2006-05-05

**Authors:** Irene Tiemann-Boege, Peter Calabrese, David M Cochran, Rebecca Sokol, Norman Arnheim

**Affiliations:** 1 Molecular and Computational Biology Program, University of Southern California, Los Angeles, California, United States of America; 2 Obstetrics, Gynecology and Medicine, and Women's Hospital, Health Sciences Campus, University of Southern California, Los Angeles, California, United States of America; University of Chicago, United States of America

## Abstract

For decades, classical crossover studies and linkage disequilibrium (LD) analysis of genomic regions suggested that human meiotic crossovers may not be randomly distributed along chromosomes but are focused instead in “hot spots.” Recent sperm typing studies provided data at very high resolution and accuracy that defined the physical limits of a number of hot spots. The data were also used to test whether patterns of LD can predict hot spot locations. These sperm typing studies focused on several small regions of the genome already known or suspected of containing a hot spot based on the presence of LD breakdown or previous experimental evidence of hot spot activity. Comparable data on target regions not specifically chosen using these two criteria is lacking but is needed to make an unbiased test of whether LD data alone can accurately predict active hot spots. We used sperm typing to estimate recombination in 17 almost contiguous ~5 kb intervals spanning 103 kb of human Chromosome 21. We found two intervals that contained new hot spots. The comparison of our data with recombination rates predicted by statistical analyses of LD showed that, overall, the two datasets corresponded well, except for one predicted hot spot that showed little crossing over. This study doubles the experimental data on recombination in men at the highest resolution and accuracy and supports the emerging genome-wide picture that recombination is localized in small regions separated by cold areas. Detailed study of one of the new hot spots revealed a sperm donor with a decrease in recombination intensity at the canonical recombination site but an increase in crossover activity nearby. This unique finding suggests that the position and intensity of hot spots may evolve by means of a concerted mechanism that maintains the overall recombination intensity in the region.

## Synopsis

Meiotic crossover events are not randomly distributed across the human genome, but are concentrated in many small regions of a few kb with high recombination rates compared to surrounding regions. How the distribution of recombination events affects the association of different alleles along the chromosome (linkage disequilibrium, or LD) was recently addressed using sperm typing in regions already known or suspected to contain unusually high recombination intensities. In the current paper, the authors used sperm typing to examine recombination in a region not known or suspected of containing recombination hot spots. They first established the crossover distribution pattern within a 103-kb region of human Chromosome 21. Then, they compared their data to predictions of crossover distributions estimated by statistical analyses of polymorphism in the region. They found a good concordance between the two, although it was not perfect. To the authors' knowledge, this work is the first to compare LD-based estimates of recombination to sperm-typing data from regions not previously known or suspected of containing recombination hot spots. In addition, one of the studied hot spots revealed an example of a decrease in recombination intensity with a concurrent increase at a nearby site. This unique observation suggests that the activity of hot spots may evolve in a concerted fashion such that the overall recombination activity of the region is maintained.

## Introduction

Recently there has been a great deal of interest in understanding how recombination varies in the human genome and how hot spots of recombination (reviewed in [1–7]) may affect patterns of linkage disequilibrium (LD). This has been an important issue, especially with regard to association studies that use LD to map and identify complex disease genes [8–11]. Considerable differences in LD among neighboring sequences divide the human genome into blocks of high LD (haplotype blocks) 5–100 kb in size [8,10,12,13]. These LD blocks can reflect the recombination history of a genomic region, although other factors such as mutation, selection, and demographic events (like migration and population bottlenecks) might also play a role [10,11,14,15].

Correlating LD patterns with crossing-over data requires measuring allelic recombination at levels of resolution and accuracy commensurate with the length of typical haplotype blocks. Genetic mapping of the human genome using pedigree analysis [16–18] cannot estimate recombination intensities with the required accuracy compared to sperm typing that can readily examine many more meioses. In one approach, large numbers of individual human sperm are analyzed [19–22] as in, for example, the studies of allelic recombination at the major histocompatibility complex (MHC), pseudoautosomal, and β-globin hot spot regions. However, the highest levels of resolution and accuracy are obtained when analyzing DNA from sperm pools [23–26]. Pooled sperm typing studies of the MHC on chromosome 6 and the minisatellite 32 (MS32) region on Chromosome 1 [23–25,27] showed allelic recombination could be localized to small areas 1–2 kb in length where crossover activity was substantially higher compared to neighboring sequences (hot spots). Regions between hot spots contained low recombination activity (0.04–0.15 cM/Mb) and were defined as “cold areas” [1,23,24,28].

Recently, in silico methods based on LD analysis were developed to predict recombination hot spots throughout the human genome [29–32]. The potential of these LD estimators to predict a hot spot was tested by comparing the location and intensity of the predicted hot spots with sperm-typing data and the DeCode pedigree map [29–32]. When the data on the MHC and MS32 region were compared to the in silico results, there existed, a good correlation between the intensity of recombination and the strength of LD [3,23,24,29–32], although with some notable exceptions [24,33].

The MHC and MS32 sperm typing studies, rather than collecting data randomly throughout the entire DNA segments, analyzed specific target regions chosen by looking for either the presence of LD breakdown or because evidence from earlier experimental studies suggested the existence of a hot spot. While this approach has provided invaluable data on individual human hot spots, it raises the question of whether the observed correlations between LD and sperm-typing data might be somewhat biased due to this method of ascertainment. In other words, selecting regions for sperm-typing studies based on the presence of LD breakdown might increase the chance that methods to infer actual recombination intensities from LD breakdown would be successful.

In order to make a less biased comparison between recombination rates measured by sperm typing and those predicted by computational approaches, we used sperm typing to study recombination across a 103-kb region on Chromosome 21 at high resolution and accuracy. This region was chosen without any consideration for its potential to harbor recombination hot spots. We gathered crossover data for 17 almost contiguous intervals ~5 kb in length. We compared our sperm typing results with three different LD-based estimators of recombination. Finally, our analysis of crossover breakpoints suggested some interesting features about the evolution of recombination hot spots.

## Results

### Recombination Intensities in a Region of Chromosome 21

**Figure 1:**
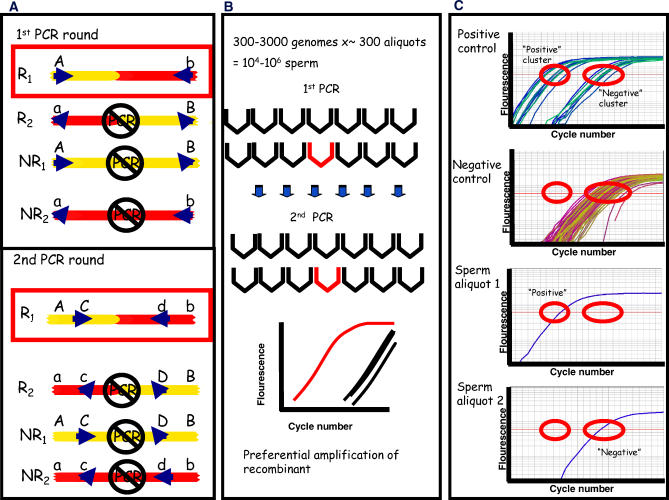
Illustration of the Experimental Approach (A) Shown here are all four possible meiotic products. One of the two recombinants (R_1_) is selectively amplified in two rounds of allele-specific PCR by using forward and the reverse primers that both form a perfect match with the chosen crossover type, in this case R_1_ (shown here in a red box). One mismatch is formed with the two non-recombinant (NR_1_ and NR_2_) meiotic products and two mismatches with the other crossover type (R_2_). In a second PCR round, new allele-specific primers anneal to an internal pair of SNPs and the specific recombinant is enriched further. (B) In order to analyze a million meioses, ~300 aliquots containing ~3,000 sperm genomes were analyzed. Given that recombination is a rare event, only a few aliquots contain a single recombinant molecule (aliquot with a single recombinant shown in red). The second PCR was performed in a real-time PCR machine to monitor the preferential amplification of the chosen recombinant over the other meiotic products. (C) The amplification curve obtained for each sperm aliquot was compared to the amplification obtained for positive controls (containing non-recombinants with, on average, one added recombinant which, based on the Poisson distribution, will render only 65% of the aliquots positive) and negative controls (containing only non-recombinants). Two distinct clusters are formed of positive and negative amplification curves. The number of sperm aliquots with positive amplification curves was considered the number of recombinants. Similarly, sperm aliquots with negative amplification curves were considered not to contain a recombinant. Additional details can be found in Materials and Methods.

Recombination was measured using allele-specific primers that selectively amplify, in two rounds of PCR, a single recombinant in the presence of an excess of non-recombinants (Figure 1). In order to monitor the preferential amplification of the specific recombinant we used a real-time thermocycler that monitored the increase of fluorescence in each cycle that is proportional to the amount of DNA produced [34]. The aliquots containing a recombinant were evaluated by comparing the amplification curves to those of positive and negative controls (see Figure 1 and Materials and Methods).

**Figure 2:**
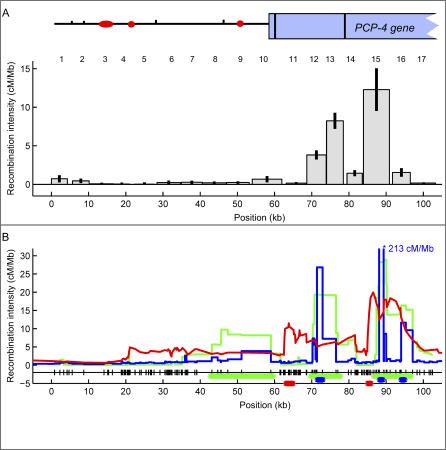
Recombination across a 103-kb Region in Chromosome 21 (A) The histogram shows the recombination intensities measured in 17 intervals (the interval number is shown on top of each bar) of the 103-kb region of Chromosome 21. For intervals 13 and 15 we plotted the average of both reciprocal crossovers. Shown on top of the histogram is the genetic structure of the region (circles/ovals represent long interspersed nuclear elements and short interspersed nuclear elements, tick marks represent long terminal repeats, and the rectangle represents the partial *PCP4* gene; exons are shown as black vertical lines). The recombination intensity in cM/Mb is corrected for false positives. Gaps represent areas where no recombination data could be collected. The 95% confidence interval for each measurement is shown as an error bar. (B) Recombination intensities calculated with three different LD estimators: LD-Hat in green, Hotspotter in blue, and ABC in red. Note that the predicted peak at ~90 kb (interval 15) for Hotspotter is 213 cM/Mb and thus off scale. All three estimators used the Perlegen SNP database [35]. SNPs are shown as marks next to the *x*-axis. The thick green, blue, or red horizontal lines above the *x*-axis mark the region with statistical support for a hot spot in LDHat, Hotspotter, or ABC, respectively. See Materials and Methods and Table 1 for details and Protocol S6 for a list of estimated recombination intensities for all three algorithms in our region.

At the time our study was initiated, a detailed single nucleotide polymorphism (SNP) map for Chromosome 21 had just been made available [12], making this chromosome an attractive target for our study. The 103-kb region we analyzed in Chromosome 21 was chosen based on a number of criteria. We sought a region with a high density of validated SNPs, a G + C content between 30% and 60%, and a variety of genetic elements typical of the genome as a whole including coding and noncoding as well as repeated sequences. We also sought a region where classical linkage data suggested recombination fractions (on a megabase [Mb] scale) to be close to the genome average [18]. The chosen region lies between SNPs rs10622653 and rs2299784 on Chromosome 21, has a SNP density of, on average, one per 220 bp, an average G+C content of 42%, and diverse genetic elements (long terminal repeats, long interspersed nuclear elements, and short interspersed nuclear elements) including, in the downstream region, two-thirds of the *PCP4* gene as shown in Figure 2A. The study region was contained within a larger 1-Mb interval having a male recombination rate of 2.4 cM/Mb as measured by classical linkage analysis [18]. Using 72 appropriately chosen SNPs, we genotyped 240 individuals, mostly of European descent. The 103-kb region was divided into 17 almost contiguous intervals, each approximately 4–8 kb in length. Between intervals, gaps totaling 8.4 kb exist for which no crossover data was gathered due to the lack of informative polymorphisms in these areas. Thus, crossover data was gathered for 94.6 kb of the 103-kb region. Recombination was measured at kb resolution by typing, on average, a million meioses (sperm genomes) per interval using an average of ~5 informative donors. For each interval, an almost equal number of genomes that could not have contained a crossover were also studied to characterize the background of the assay (see Materials and Methods).

**Table 1:**
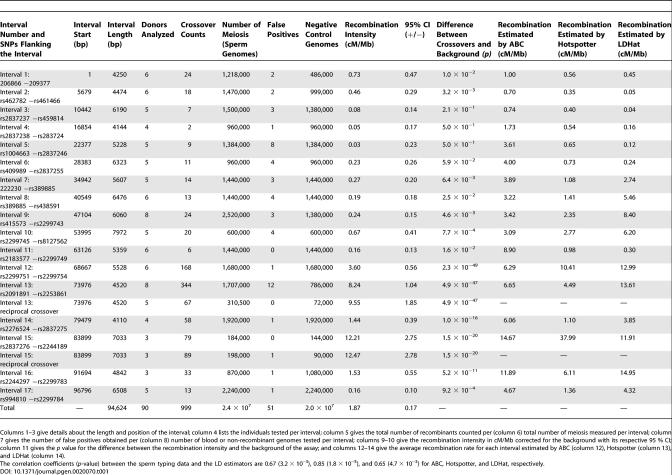
Recombination Measurements Obtained for Each of the 17 Intervals and for the Reciprocal Crossover Products for Intervals 13 and 15

Recombination occurred at different intensities throughout the 103-kb region. All but four of the intervals had intensities above the assay background ranging from 0.16 cM/Mb (95% confidence interval: 0.06, 0.26) to 12.47 cM/Mb (9.69, 15.25) as seen in Figure 2A and Table 1. The overall crossover activity was estimated to be 1.87 cM/Mb (1.70, 2.04), which is comparable to 2.4 cM/Mb estimated by pedigree analysis for the male recombination intensity in the 1-Mb segment that includes our 103-kb region [18]. Two intervals, covering ~12% of the total analyzed sequence, had recombination hot spot activity accounting for 71% of the total crossovers measured. The average of the reciprocal crossovers in interval 13 (position ~74–78 kb) was 2.5–6.2-fold higher and the average of both reciprocals in interval 15 (position ~84–91 kb) was 8.0–8.6 times greater than their respective neighboring intervals. In the case of interval 13, recombination intensity estimates were virtually the same for both reciprocal crossovers, 8.24 cM/Mb (7.20, 9.28) and 9.55 cM/Mb (7.70, 11.40), respectively. Likewise, for interval 15, the estimates were also similar, 12.21 cM/Mb (9.46, 14.96) for one and 12.47 (9.68, 15.25) for the other reciprocal product. Intervals 13 and 15 lay within introns 1 and 2 of the *PCP4* gene, respectively.

### Recombination Intensities Compared to Data on LD

Figure 2B compares the recombination frequencies measured by sperm typing with estimates from three different algorithms of LD analysis (LDHat [30], Hotspotter [32], and ABC; see Materials and Methods). For each algorithm, the recombination rates were estimated separately for each of the three populations in the Perlegen SNP database [35], and then these population estimates were averaged. All three algorithms inferred elevated recombination activity in interval 15, one of the regions determined to be hot by sperm typing. LDHot [29], a hypothesis-testing algorithm with methodology similar to LDHat, determined that this peak was significant. Examining the 95% credibility regions from Hotspotter and ABC also gave statistical support for this hot spot (see Materials and Methods). Hotspotter also estimated an elevated recombination activity 5 kb downstream from interval 15. The wider peaks inferred by the other two algorithms that covered interval 15 overlapped this region. Sperm typing did not show significantly elevated recombination intensity in the remaining downstream intervals.

All three algorithms also estimated increased recombination around interval 13, the other hot region found by sperm typing. LDHat predicted a 10-kb region covering intervals 12 and 13 with significantly elevated recombination estimates. For Hotspotter, the peak was located in interval 12, and was statistically supported. In comparison, ABC estimated increased recombination ranging from interval 11 to interval 13, which also had statistical support for a region in interval 11.

A third significant hot spot was inferred by LDHot at around 50 kb. All the algorithms estimated some recombination activity in this area but not as high as the regions previously discussed. In contrast, sperm typing did not observe an elevated rate in this area. All three methods estimated little recombination activity from 0–40 kb, which is in agreement with sperm-typing measurements.

Using the three algorithms of LD analysis, we also estimated recombination using only the European-American SNP data of Perlegen [35] or HapMap [36] (see Protocol S1), since this population is most similar to the sperm-typing donors. For Hotspotter, LDHat, and ABC, the positions of elevated recombination rates were similar for the averaged population and each of the individual European-American populations (though the heights of the peaks differed).

### Crossover Distributions in Intervals 15 and 13

**Figure 3:**
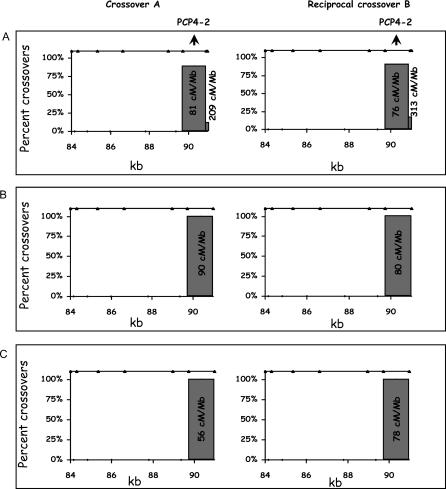
Crossover Distribution in Interval 15 for Three Different Individuals The breakpoint of the crossover was determined by genotyping the SNPs (shown as triangles on top of each graph) using PCR product obtained from individual crossovers. Percentages represent the fraction of crossovers counted between two adjacent informative SNPs and are plotted against the position of the crossover for both type A and the reciprocal type B recombinants. The numbers associated with each bar represent the recombination intensities in cM/Mb (see details in Protocol S2). (A) Crossover distribution in interval 15 for an individual based on 36 and 24 recombinants recovered from 66,000 and 44,000 meioses, respectively. Note that this individual has one more informative SNP 50 bp before the end of the interval compared to the other two donors. Only a small fraction of the crossovers occur within these last 50 bps, but the estimated recombination intensity ranges between ~200 to 300 cM/Mb because this region is so small. (B) Crossover distribution in a second donor based on 37 and 12 recombinants recovered from 66,000 and 24,000 meioses, respectively. (C) Crossover distribution for a third donor based on 23 and 43 recombinants recovered from 66,000 and 88,000 meioses, respectively. For a detailed comparison of the crossover distribution with the three LD based estimates, see Protocol S5.

We analyzed the distribution of crossover breakpoints in the two hot spot–containing intervals for both of the reciprocal recombinants to identify the specific location of crossing over. PCR products from individual crossover events were genotyped using SNPs internal to those used for allele-specific amplification. The resolution of breakpoint identification depends on the density of informative SNPs within an interval and the availability of sperm samples from individuals informative for each SNP. For interval 15, all of the crossovers in the three individuals studied were concentrated between positions 89.6–90.9 kb (Figure 3). This subinterval defined the hot spot PCP4–2. The intensity of recombination for the 3 different donors averaged 78.9 cM/Mb (68.9, 88.9) within this 1-kb subinterval (see Protocol S2). The hot spot could extend into the 1-kb gap between intervals 15 and 16 but it is unlikely to extend much further since interval 16 had an 8-fold lower recombination activity than interval 15. Thus, the total length of the PCP4–2 hot spot is unlikely to be greater than 2 kb.

**Figure 4:**
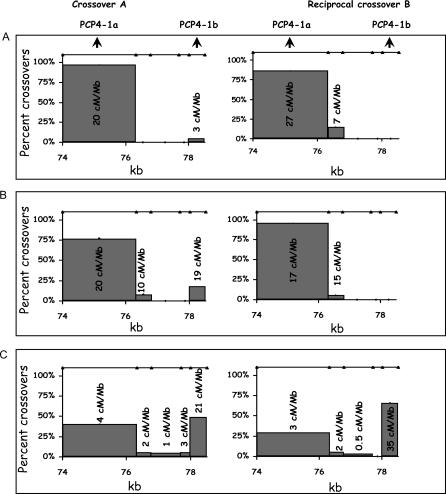
Crossover Distribution in Interval 13 for Three Different Individuals For additional details see the legend to Figure 3. (A) Crossover distribution for a donor based on 30 and 21 reciprocal recombinants recovered from 126,000 and 89,300 meioses, respectively. Numbers associated with each bar are the recombination intensities in cM/Mb. (B) Crossover distribution for a second donor based on 13 and 20 recombinants recovered from 42,000 and 60,400 meioses, respectively. (C) Crossover distribution for a third individual based on 25 and 49 recombinants recovered from 223,200 and 358,000 meioses, respectively. See Protocol S5 for a comparison of averaged recombination intensities of the 3 donors with the LD estimators.

In interval 13, more than 90% of the crossovers measured using two donors were clustered in the region between 74–76.3 kb (see Figures 4A and 4B). The average recombination intensity including both reciprocals was 21.1 cM/Mb (17.1, 25.2). Characterization of a third donor led to a completely unexpected result. The majority of crossovers (59%) within interval 13 were shifted to position 78–78.5 kb (Figure 4C), thereby defining two active crossover regions: PCP4-1a (74–76.3 kb) and PCP4-1b (78–78.5 kb). Compared to the average of the first two donors, the third individual had a ~6-fold reduction of crossover activity at PCP4-1a (from 20.9 cM/Mb to 3.6 cM/Mb, *p* = 5 × 10^−12^) but had a ~8-fold increase at PCP4-1b (from 3.5 to 28.1 cM/Mb, *p* = 2 × 10^−7^). When interval 13 is considered as a whole, the recombination intensity averaged over the first two individuals is ~2-fold higher than the third donor (from 10.6 cM/Mb to 5.1 cM/Mb, *p* = 2 × 10^−5^).

## Discussion

Our study of 23.5 million informative meioses demonstrates highly localized (1–2 kb) active hot spots of recombination in regions not previously known or suspected to contain hot spot activity. We estimate that 71% of the recombination occurred in ~12% of our sequence. This experimental value is comparable to those based on LD estimates suggesting that 80% of crossovers occur in 10%–20% of the total sequence in the case of both Chromosome 21 as well as the whole genome [29].

Our results provide additional data on recombination in so-called cold areas. Our estimate of recombination intensity excluding hot spot intervals 13 and 15 (as well as adjacent intervals 12, 14, and 16) averaged 0.37 cM/Mb (0.27, 0.47) based on 132 recombinants from 12.3 million meioses. This value is anywhere from 2–10-fold higher than the estimate of 0.04–0.15 cM/Mb for the MHC cold regions (based on LD analysis [23]) or 0.04 cM/Mb for the MS32 region [24]. This latter study, however, concentrated on collecting data from the hot spots and thus, the sample size of crossovers in cold spots was minimal [24]. Accurate estimates of recombination in cold areas may prove useful in modeling the role of crossing over during chromosomal evolution [29–32,37–42]. Moreover, using variable recombination estimates, validated by sperm-typing data, should improve the performance of fine-scale mapping algorithms [38,39].

We searched our 103-kb chromosomal segment for promising DNA sequence motifs suggested to be correlated with the location of human hot spots predicted by LD analysis [29]. Notably, two copies of the CCCCACCC octamer motif were found in our 103-kb region. Both were located in interval 13, one in PCP4-1a, and the other in PCP4-1b. The CCTCCCT heptamer motif, which may drive hot spot activity in men polymorphic for these motifs [29], was found 21 times in the study region. One was close to the PCP4–2 hot spot. A THE1A/B retrotransposon that is found in 2%–3% of the hot spots predicted by LD was present twice in our segment at position 1.6 kb and 43.9 kb, but the sequence was truncated. A complete list of other motifs reported to be enriched in hot spots compared to cold areas [29] and that are present in our 103-kb region can be found in Protocol S3.

Our in silico analysis, in general, showed a good correspondence between the recombination activity measured by sperm typing and that predicted by LD data. However, the correspondence is not perfect, and there are several possible reasons for this. Even when all the modeling assumptions are correct, we believe it is unclear how well the algorithms perform. All the algorithms assume that at a given position in the genome, the recombination rate is constant throughout time and across individuals in that population. The LD methods estimate historical recombination rates; thus, a hot spot that was present in the past but has now been lost would result in an elevated LD-based estimate which would disagree with sperm typing measurements [24,28,33]. Likewise, if a new hot spot has recently emerged, it would only leave a weak signal in the LD data, while it would be observed by sperm typing [24,28,33]. As suggested by our findings in interval 13, another possible violation of the modeling assumptions would be when a hot spot changes its position, so that the hot spot has a different position in different individuals. Furthermore, a hot spot might be present in some individuals but not others. This heterogeneity among individuals might or might not be sex specific. Further, hot spots may also be population specific as reported by [31]. If a hot spot is polymorphic in the population, it will leave a weaker signal in the LD patterns than if this hot spot was fixed, but the quantitative details of this and all the other violations of the modeling assumptions are unknown. A hot spot that is polymorphic in the population will only be observed by sperm typing if the appropriate subset of the population is sampled. Therefore, it is possible that a region that is estimated to have elevated recombination activity by the LD methods, but for which no such elevation is measured by sperm typing, is (1) a historical hot spot which is no longer active [24,28,33]; or (2) a hot spot that is polymorphic in the population, and for which not enough individuals were typed to include a donor with this hot spot. Likewise, if sperm typing measures a hot spot in a region which is not supported by the LD methods, it is possible that (1) this hot spot has only recently emerged [24,28,33]; or (2) it is polymorphic in the population.

Based on the recent genome-wide analyses of recombination using LD data, evidence for a hot spot seems to exist on average every 50–200 kb [29–31]. Genome-wide differences in the patterns of hot spot distribution between humans and chimpanzees computed using LD data [40–43] suggests that hot spots may vary in position over evolutionary time [24,29,30]. An ongoing birth and death of hot spots during recent human evolutionary history is also suggested by LD data from different populations producing different hot spot distributions [31]. Consistent with this idea are both low-resolution [3,20,44] and high-resolution [24,28,33] sperm-typing studies showing variation in recombination intensity among individuals for the same DNA segment.

Our understanding of recombination hot spots is limited not only with regard to the molecular basis for their activity (see [1,2,6]) but also in regard to their birth and death during evolution. We define hot spots as DNA segments that, in some as yet unknown way, help direct the position and frequency of crossing over during meiosis. Based on what we know about hot spots in yeast it has been proposed that, once a hot spot arises, it is destined to be eliminated [45]. Hot spot alleles on one homolog that promote double-strand break (DSB) formation in their vicinity (in *cis*) will be preferentially converted to a less-active allele present on the other homolog during DSB repair, eventually leading to the loss of the hot spot allele in the population. A number of computational studies have considered what factors (e.g., mutation, selection, crossover interference, and hot spot competition) might play a role in determining the rate that hot spots are eliminated under this model (reviewed in [45]).

All hot spots may not necessarily evolve according to the mechanistic assumptions of the model described above. A recent study has shown that meiotic DSBs in the yeast Schizosaccharomyces pombe may be detected at significant distances from the site of a well-defined hot spot sequence, and it was suggested that the rate of hot spot loss would be reduced in proportion to the distance between the hot spot and the DSB site [46]. This is because the hot spot region on the initiating chromatid must be included within the conversion tract if it is to be converted, and the probability of inclusion decreases with increasing distance between the hot spot and the DSB. Furthermore, and as suggested by a reviewer, an allelic variant of a particular hot spot with the property of directing DSB formation to distant sites might increase in frequency in the population. Consider individuals heterozygous for this “displacing” hot spot allele that initiates DSBs at a distant site and a “normal” hot spot allele that initiates DSBs nearby. When the displacing allele initiates a DSB, it would rarely be converted to the normal allele. Yet the displacing allele would be more likely to be the donor for DSB repair when a break was initiated by the normal allele.

Another view concerning the birth and death of hot spots is suggested by our study of interval 13. The alteration in crossover distribution in one of three individuals was described as a decrease in recombination intensity at PCP4-1a with a simultaneous increase in PCP4-1b that together resulted in only a relatively small overall change (2-fold) in recombination intensity for the interval as a whole. These findings might suggest that, rather than PCP4-1a and PCP4-1b being two tightly linked hot spots evolving independently of one another [47], recombination intensity and position over the whole interval might change in some concerted fashion such that the overall recombination activity of the region remains similar. One possible mechanism for such a concerted change involves competition between adjacent hot spots as has been well documented in yeast (reviewed in [45]). If an allele arose with a partially inactivating mutation affecting the dominant member of a hot spot pair, an adjacent, but previously suppressed, hot spot might show significantly increased activity. Our ability to predict the kinds of mutation that can alter hot spot activity is limited given that we do not really understand how hot spots function at the molecular level, although mutations affecting chromatin structure in the hot spot region are likely to be important (see [2]). Such mutations might also explain alterations in crossover position and intensity even in a region with only a single hot spot if changing chromatin structure modified the position of DSB initiation.

Finally, it is also been proposed [5] that hot spots define a local region where DSBs may take place but the exact position of a crossover may be determined by an epigenetic process with the outcome differing among individuals. Regardless, variation among individuals in the patterns of crossover distribution in the same local region may represent a transition stage in hot spot evolution, presaging a shift in hot spot position and intensity along the chromosome.

## Materials and Methods

### Samples.

Semen was obtained from 195 anonymous donors and semen and blood samples from the same donor were also collected from 45 additional individuals according to protocols approved by the Institutional Review Board of the University of Southern California. Donors were mainly of European descent. Sperm DNA was extracted using Puregene DNA Isolation Kits (Gentra Systems, Minneapolis, Minnesota, United States) with the addition of 40 μM DTT during the cell lysis step. Blood was extracted using PAXgene blood DNA kit (Qiagen, Valencia, California, United States).

### Genotyping.

Publicly available database SNPs were chosen from a ~103-kb region located between SNPs rs10622653 and rs2299784. Genotypes were determined by allele-specific PCR performed in a real-time PCR machine (GeneAmp 5700 PE, Applied Biosystems, Foster City, California, United States; or Opticon 2, MJ Research, Waltham, Massachusetts, United States). Allele-specific primers were designed to form a perfect match with one allele but not with the alternative allele. The last 4 phosphodiester bonds at the 3′ end were substituted by phosphorothioate bonds to increase allele-specific selectivity. The PCR buffer contained 1× Buffer Gold (Applied Biosystems), 2 mM MgCl_2_, 0.16 mM dNTPs, 0.4 uM forward and 0.4 uM reverse allele-specific primers (with 4 phosphorothioate bonds at the 3′ end), 0.1× SYBR Green I (Molecular Probes, Eugene, Oregon, United States), 1 U per reaction of Taq Gold or 5 U per reaction of rdZ05 Gold (Roche, Basel, Switzerland) and 10 ng DNA. For each SNP two aliquots were amplified, one for each allele. For every sample, the difference between the Cts (value at which the amplification signal reaches a certain threshold value during logarithmic accumulation of PCR product) of the two allele-specific reactions was used to determine the genotype of the sample. A 4–5 or more Ct difference indicated homozygosity for the allele with lower Ct value. A difference of 0 or 1 was taken to indicate heterozygosity. Samples with Ct differences outside these values were genotyped again. The genotype, sample ID, Ct difference, and the amplification graphs were stored in an Access (Microsoft Office) database.

### Haplotyping.

The phase of two exterior and two interior SNPs flanking each interval is required for sperm typing. Samples heterozygous at all four SNPs were amplified with the Expand Long Template PCR System (Roche) using allele-specific primers for the exterior SNPs. Four reactions were set up, each containing the forward and reverse allele-specific primers that perfectly matched one of the four possible haplotypes. The haplotype for the exterior SNP pair was determined based on which of the four reactions amplified first. The phase for the internal SNP pair was obtained by genotyping a 100,000 dilution of the haplotyping reactions. Details for haplotyping are found in Protocol S4.

### Counting recombinants.

First the number of amplifiable genomes per nanogram of DNA for each recombination interval was calculated (see Protocol S4B) by using real-time PCR with primers outside the outermost flanking pair of SNPs for the interval. The amplifiable sperm DNA was quantitated by comparison with a standard series of 100, 30, 10, and 3 nanograms of high-molecular-weight genomic DNA (BD Biosciences, San Diego, California, United States).

To measure crossing over, sperm DNA from an informative individual was divided into ~20–80 aliquots each containing 300, 1,000, or 3,000 genomes. An equal number of aliquots containing 300, 1,000, or 3,000 genomes of blood DNA from the same individual (or in some cases a mixture of sperm DNA from both non-recombinant haplotypes) were also included in the same experiment (negative controls). As a positive control, 20 aliquots with an average of a single recombinant molecule in 300, 1,000, or 3,000 non-recombinant genomes per reaction were used. Every PCR reaction contained 0.4 μM of each of the appropriate forward and reverse allele-specific primer, which had two to four phosphorothioate bonds and sometimes mismatches at the 3′ end. The PCR reaction also contained 1× Expand Long Template buffer 2 or 3, 0.5 mM dNTPs, and 0.75 U of enzyme per reaction (Expand Long Template PCR System; Roche). Only the second-round PCR included 0.1× SYBR Green I (Molecular Probes). The first round of PCR was set up in a separate biosafety cabinet irradiated beforehand with UV light and carried out using a DNA Engine Tetrad 2 (MJ Research). A 0.5 μl aliquot of the first-round amplification product was added to the second round and amplified using a 7900 HT Real-Time PCR System (Applied Biosystems). Both first- and second-round PCR reactions were performed in 384-well plates in volumes of 10 μl.

Positive and negative controls were used to determine the efficiency and specificity of each PCR reaction. Only ~65% of the positive controls (each containing on average one recombinant molecule) were expected to generate a positive signal (defining the positive cluster of amplification curves) based on the Poisson distribution of single-molecule dilution [48,49]. For all experiments we obtained the expected number of positives, and thus can assume that we did not underestimate the number of crossovers. The negative controls defined the negative cluster. Optimally, the positive cluster was easily distinguishable from the negative cluster with a gap between the two of four to five Cts, although a difference of approximately two cycles was enough to distinguish the two clusters. In the case when there was an overlap of the two clusters, the experiment was discarded. The recombinant count was the number of sperm aliquots that fell within the positive cluster. In order to ensure that each positive reaction contained only one recombinant molecule, we adjusted the number of sperm genomes per aliquot such that the total fraction of positive reactions per experiment would be less than 30%.

We also controlled for false positives arising due to technical artifacts such as the amplification of a non-recombinant by the misextension of the allele-specific primers [50,51] or the extension, by truncated PCR products, of the reciprocal non-recombinants. This is a problem especially for regions with very low recombination frequencies because technical artifacts, although rare, could produce as many positives as a sample with very few recombinants, lowering the accuracy of the recombination measurement. The negative controls were composed of DNA that did not contain a recombinant. For this purpose we used the blood genomes from the donor or, if this was not available, a mixture of both non-recombinant haplotypes from other donors' sperm DNA. In both cases we typed the same number of negative controls as the number of typed sperm genomes for each interval (on average a million genomes per interval). In the case when a negative fell within the positive cluster, it was called a false positive and was used to estimate the background signal. The recombination intensity for each interval was estimated by subtracting the fraction of false positives from the fraction of crossovers. To convert this corrected crossover frequency to cM/Mb, we multiplied the corrected crossover frequency by two (to consider that we usually measured only one of the two reciprocal products for each interval), multiplied by 100, and then divided by the interval length in Mb. Confidence intervals for each DNA interval were calculated by the standard Poisson approximation. Based on the nature of the assay, the specificity or number of false negatives varied for each interval. The specificity mainly depends on the sequence context surrounding the 3′ end of the allele-specific primer. The specificity also depends on the optimization time invested in each interval.

### Estimating patterns of LD.

For Figure 2B, we used the Perlegen dataset [35]. This dataset contains three populations; we estimated the recombination rates separately for the three populations, and then for each method, we averaged these population estimates. In order to change units from the population genetics ρ (rho) to cM/Mb we used an effective population size of 15,700 for the African-American population, and 10,000 for the European-American and Han Chinese populations [29]. All the algorithms were run on a 140-kb sequence that included the 103 kb of interest plus an additional 20 kb on either side to minimize any boundary effects. In Protocol S1, we also show the estimates computed using the Perlegen European-American sample or the HapMap [36] European-American sample.

We used three methods to infer recombination rates from the LD data: LDHat (v2.0) [30], Hotspotter (PHASE v2s.1.1) [32], and ABC (P. Calabrese, unpublished data). LDHot [29] is a hypothesis-testing algorithm with methodology similar to LDHat. The results shown in Figure 2B for LDHat and LDHot were previously published by others as supplementary materials [29]; those shown in Protocol S1 are our own implementation. We ran LDHat with the suggested parameters: block penalties of 5 and 20, an initial guess of 56 for 4Neρ (which is 0.4 times the region width in kb, as suggested by [29,30]), 10 million iterations; we sampled every 2,000 iterations and ignored the first one-third of the iterations. We also tried different starting values and longer runs; none of these changes, nor the different block penalties of 5 and 20, had much effect on the estimates.

We ran Hotspotter (PHASE v2s.1.1) with the suggested parameters. In order to test for statistical significance, we observed whether the estimates exceeded the 95% credibility regions of the flanking regions. We did this separately for each population, and only reported a region to be significant if it was significantly elevated in at least two of the three populations.

The ABC method is an Approximate Bayesian Computation method [52]. Briefly, the coalescent process is simulated with the recombination parameter chosen from a prior distribution. Numerous summary statistics are calculated. If these statistics are sufficiently close to the statistics from the actual data then the recombination parameter is accepted; otherwise, it is rejected. This procedure is repeated many times and the accepted parameters form the approximate posterior distribution. The novel part of this procedure is the metric on the collection of summary statistics. We tested for statistical significance exactly as we did for the Hotspotter algorithm.

## Supporting Information

Protocol S1 European LD Map (41 KB PDF)

Protocol S2 Crossover Distribution for Intervals 13 and 15 (36 KB PDF)

Protocol S3 Motif Table (22 KB PDF)

Protocol S4 PCR Conditions for Measuring the Number of Crossovers and Amplifiable Genomes (68 KB PDF)

Protocol S5 Comparison of LD and Sperm Typing in Intervals 13 and 15 (43 KB PDF)

Protocol S6 List of Recombination Intensities for the LD Estimators (30 KB XLS)

### Accession Number

The GeneBank (http://www.ncbi.nlm.nih.gov) accession number for the *PCP4* gene is NM 006198.

## Abbreviations

DSB: double-strand break
LD: linkage disequilibrium
MHC: major histocompatibility complex
MS32: minisatellite 32
SNP: single nucleotide polymorphism
