# Supplementary material for: High-Resolution Recombination Patterns in a Region of Human Chromosome 21 Measured by Sperm Typing
Source: PLoS Genet. 2006 May 5;2(5):e70. doi: 10.1371/journal.pgen.0020070 (PMC1456319; doi:10.1371/journal.pgen.0020070)

Protocol S1- European LD map. Recombination intensities measured by sperm typing as shown in manuscript Figure 2 (panel A) compared to the intensities calculated with three different LD estimators. The estimators used the Perlegen SNP database (panel B) or the Hap Map SNP database (panel C) for the European American population. LD-Hat is shown in green, Hotspotter in blue, and ABC in red. Note that some of the peaks at ~70 kb, ~90 kb and ~95 kb are off scale.

A

B

C

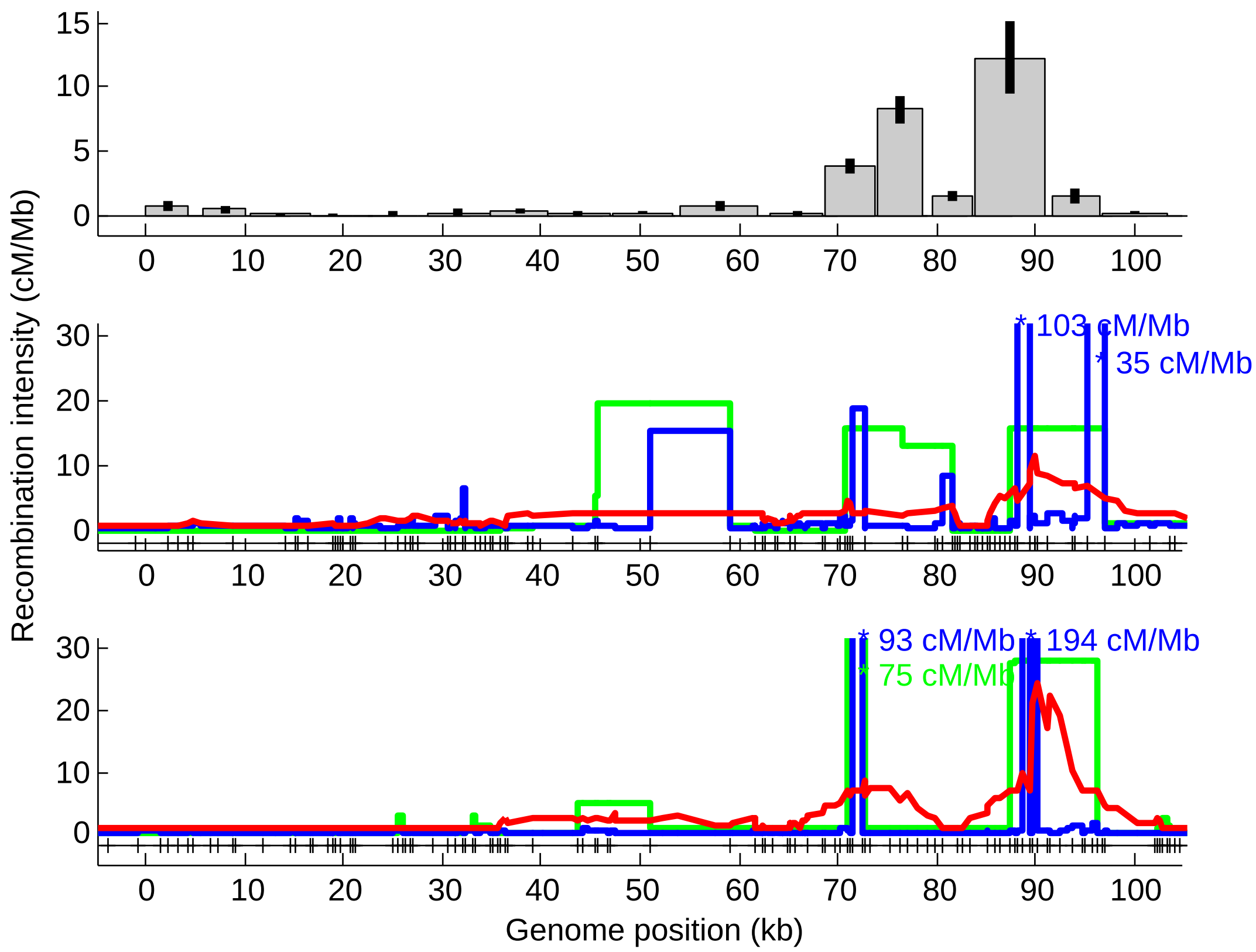

Supplement: Supplementary file 1 [file pgen.0020070.sd001.pdf]
