# Supplementary material for: High-Resolution Recombination Patterns in a Region of Human Chromosome 21 Measured by Sperm Typing
Source: PLoS Genet. 2006 May 5;2(5):e70. doi: 10.1371/journal.pgen.0020070 (PMC1456319; doi:10.1371/journal.pgen.0020070)
Supplement: Supplementary file 2 [file pgen.0020070.sd002.pdf]

Protocol S2 -Crossover distribution for Interval 13 and 15. Shown are the recombinant haplotypes, where black and red are the different parental alleles and the capital letters represent the SNPs used for the second round allele-specific amplification (column 1); the typed SNP, where the first and the last SNP are the ones used for allele-specific amplification (column 2); the number of crossovers between two adjacent SNPs, eg. 29 crossovers between rs2091891 and rs11088498, (column 3); the fraction of crossovers per total recombinants (column 4); the recombination intensity, which is crossovers per total meioses analyzed, times 2 (to account for both reciprocals), then times one hundred and divided by the distance between two adjacent SNPs in Mb (column 5); the distance between adjacent SNPs (column 6); and the position of the SNP in the 103 kb region studied (column 7).

Crossover distribution for Interval 13

Individual 5139 **Type A**

	SNP	# of crossovers	% recombinants	cm/Mb	fragment size	Position
Start	rs2091891					73976
G-a-c-t-t-T	rs11088498	29	97%	19.70	2328	76304
G-g-c-t-t-T	rs11702550	0	0%	0.00	459	76763
G-g-t-t-t-T	rs1006891	0	0%	0.00	919	77682
G-g-t-c-t-T	rs1006890	0	0%	0.00	307	77989
G-g-t-c-c-T	rs2253861	1	3%	3.13	507	78496
	Total	30	Total meiosis analyzed		126,000	

Individual 5139 **Type B**

	SNP	# of crossovers	% recombinants	cm/Mb	fragment size	Position
Start	rs2091891					73976
C-g-t-c-c-C	rs11088498	18	86%	27.01	2328	76304
C-a-t-c-c-C	rs11702550	3	14%	7.21	459	76763
C-a-c-c-c-C	rs1006891	0	0%	0.00	919	77682
C-a-c-t-c-C	rs1006890	0	0%	0.00	307	77989
C-a-c-t-t-C	rs2253861	0	0%	0.00	507	78496
	Total	21	Total meiosis analyzed		89,300	

Individual 5141 **Type A**

	SNP	# of crossovers	% recombinants	cm/Mb	fragment size	Position
Start	rs2091891					73976
G-a-c-t-t-T	rs11088498	10	77%	20.45	2328	76304
G-g-c-t-t-T	rs11702550	1	8%	10.37	459	76763
G-g-t-t-t-T	rs1006891	0	0%	0.00	919	77682
G-g-t-c-t-T	rs1006890	0	0%	0.00	307	77989
G-g-t-c-c-T	rs2253861	2	15%	18.78	507	78496
	Total	13	Total meiosis analyzed		42,000	

Individual 5141 **Type B**

	SNP	# of crossovers	% recombinants	cm/Mb	fragment size	Position
Start	rs2091891					73976
C-g-t-c-c-C	rs11088498	19	95%	17.30	2328	76304
C-a-t-c-c-C	rs11702550	1	5%	14.63	459	76763
C-a-c-c-c-C	rs1006891	0	0%	0.00	919	77682
C-a-c-t-c-C	rs1006890	0	0%	0.00	307	77989
C-a-c-t-t-C	rs2253861	0	0%	0.00	507	78496
	Total	20	Total meiosis analyzed		60,400	

Individual 7010 **Type A**

	SNP	# of crossovers	% recombinants	cm/Mb	fragment size	Position
Start	rs2091891					73976
G-a-c-t-t-T	rs11088498	10	40%	3.8	2328	76304
G-g-c-t-t-T	rs11702550	1	4%	2.0	459	76763
G-g-t-t-t-T	rs1006891	1	4%	1.0	919	77682
G-g-t-c-t-T	rs1006890	1	4%	2.9	307	77989
G-g-t-c-c-T	rs2253861	12	48%	21.2	507	78496
	Total	25	Total meiosis analyzed		223,200	

Individual 7010 **Type B**

	SNP	# of crossovers	% recombinants	cm/Mb	fragment size	Position
Start	rs2091891					73976
C-g-t-c-c-C	rs11088498	14	29%	3.4	2328	76304
C-a-t-c-c-C	rs11702550	2	4%	2.4	459	76763
C-a-c-c-c-C	rs1006891	1	2%	0.6	919	77682
C-a-c-t-c-C	rs1006890	0	0%	0.0	307	77989
C-a-c-t-t-C	rs2253861	32	65%	35.0	507	78496
	Total	49	Total meiosis analyzed		358,000	

Crossover distribution for Interval 15

Individual 7006 **Type A**

	SNP	# of crossovers	% recombinants	cm/Mb	fragment size	Position
Start	rs2837276					83899
T-a-a-a-t-a-g-C	rs3787931	0	0%	0.0	318	84217
T-g-a-a-t-a-g-C	rs8127700	0	0%	0.0	1019	85236
T-g-g-a-t-a-g-C	rs2837282	0	0%	0.0	1297	86533
T-g-g-g-t-a-g-C	rs2837286	0	0%	0.0	2342	88875
T-g-g-g-c-a-g-C	rs2244084	0	0%	0.0	759	89634
T-g-g-g-c-g-g-C	rs2244188	32	89%	81.1	1195	90829
T-g-g-g-c-g-a-C	rs2244189	4	11%	209.0	58	90887
	Total	36	Total meiosis analyzed		66,000	

Individual 7006 **Type B**

	SNP	# of crossovers	% recombinants	cm/Mb	fragment size	Position
Start	rs2837276					83899
G-g-g-g-c-g-a-T	rs3787931	0	0%	0.0	318	84217
G-a-g-g-c-g-a-T	rs8127700	0	0%	0.0	1019	85236
G-a-a-g-c-g-a-T	rs2837282	0	0%	0.0	1297	86533
G-a-a-a-c-g-a-T	rs2837286	0	0%	0.0	2342	88875
G-a-a-a-t-g-a-T	rs2244084	0	0%	0.0	759	89634
G-a-a-a-t-a-a-T	rs2244188	20	83%	76.1	1195	90829
G-a-a-a-t-a-g-T	rs2244189	4	17%	313.5	58	90887
	Total	24	Total meiosis analyzed		44,000	

Individual 5088 **Type A**

	SNP	# of crossovers	% recombinants	cm/Mb	fragment size	Position
Start	rs2837276					83899
T-a-a-a-t-a- C	rs3787931	0	0%	0.0	318	84217
T-g-a-a-t-a- C	rs8127700	0	0%	0.0	1019	85236
T-g-g-a-t-a- C	rs2837282	0	0%	0.0	1297	86533
T-g-g-g-t-a- C	rs2837286	0	0%	0.0	2342	88875
T-g-g-g-c-a- C	rs2244084	0	0%	0.0	759	89634
T-g-g-g-c-g- C	rs2244189	37	100%	89.5	1253	90887
	Total	37	Total meiosis analyzed		66,000	

Individual 5088 **Type B**

	SNP	# of crossovers	% recombinants	cm/Mb	fragment size	Position
Start	rs2837276					83899
G-g-g-g-c-g- T	rs3787931	0	0%	0.0	318	84217
G-a-g-g-c-g- T	rs8127700	0	0%	0.0	1019	85236
G-a-a-g-c-g- T	rs2837282	0	0%	0.0	1297	86533
G-a-a-a-c-g- T	rs2837286	0	0%	0.0	2342	88875
G-a-a-a-t-g- T	rs2244084	0	0%	0.0	759	89634
G-a-a-a-t-a- T	rs2244189	12	100%	79.8	1253	90887
	Total	12	Total meiosis analyzed		24,000	

Individual 5023 **Type A**

		SNP	# of crossovers	% recombinants	cm/Mb	fragment size	Position
Start		rs2837276					83899
T-a-a-a-t-a-	C	rs3787931	0	0%	0.0	318	84217
T-g-a-a-t-a-	C	rs8127700	0	0%	0.0	1019	85236
T-g-g-a-t-a-	C	rs2837282	0	0%	0.0	1297	86533
T-g-g-g-t-a-	C	rs2837286	0	0%	0.0	2342	88875
T-g-g-g-c-a-	C	rs2244084	0	0%	0.0	759	89634
T-g-g-g-c-g-	C	rs2244189	23	100%	55.6	1253	90887
		Total	23	Total meiosis analyzed		66,000	

Individual 5023 **Type B**

		SNP	# of crossovers	% recombinants	cm/Mb	fragment size	Position
Start		rs2837276					83899
G-g-g-g-c-g-	T	rs3787931	0	0%	0.0	318	84217
G-a-g-g-c-g-	T	rs8127700	0	0%	0.0	1019	85236
G-a-a-g-c-g-	T	rs2837282	0	0%	0.0	1297	86533
G-a-a-a-c-g-	T	rs2837286	0	0%	0.0	2342	88875
G-a-a-a-t-g-	T	rs2244084	0	0%	0.0	759	89634
G-a-a-a-t-a-	T	rs2244189	43	100%	78.0	1253	90887
		Total	43	Total meiosis analyzed		88,000	
