# Supplementary material for: High-Resolution Recombination Patterns in a Region of Human Chromosome 21 Measured by Sperm Typing
Source: PLoS Genet. 2006 May 5;2(5):e70. doi: 10.1371/journal.pgen.0020070 (PMC1456319; doi:10.1371/journal.pgen.0020070)
Supplement: Supplementary file 3 [file pgen.0020070.sd003.pdf]

Protocol S3-Motif table. Sequence motifs found to be represented in the 103 kb region analyzed by sperm typing. These motifs were overrepresented in hot spots compared to cold areas as described by Myers et al. Shown here are the sequence of the motif (column 1), the number of times it was found in the 103 kb region analyzed by sperm typing (column 2), how many were within the PCP4-1a, PCP4-1b or PCP4-2 hot spots (column 3), and the specific hot spot harboring the motif (column 4).

Motif	Number of times motif is found in the 103kb region	Number of times motif is present in hot spots (kb)	Details
GAAAAAAAA	6	1	PCP4-1a
AAAAAAAAA	50	0	
CCTCCCTG	3	0	
CCCCACCC	2	2	1 in PCP4-1a and 1 in PCP41-b
CCCACCCC	1	0	
CCTCCTCT	2	0	
TCCTCCCT	6	0	
CCTCCCT	21	1	PCP4-2
CCTTCCC	21	0	
CTCCTCC	20	0	
TCCCCAG	15	0	
CCCCACC	8	1	PCP4-1a
GGGGGT	9	0	
CCCCCT	24	1	PCP4-2
CCCCCA	24	0	
CCCTGC	32	0	
CCCCAG	39	1	PCP4-1a
CCCCC	50	2	1 in PCP4-1a and 1 in PCP4-2
CCCCG	28	1	PCP4-1a
GGCCC	50	4	3 in PCP4a and 1 in PCP4-1b
GCCCC	50	2	1 in PCP4a and 1 in PCP4-1b
CCCGC	24	0	
