# Supplementary material for: High-Resolution Recombination Patterns in a Region of Human Chromosome 21 Measured by Sperm Typing
Source: PLoS Genet. 2006 May 5;2(5):e70. doi: 10.1371/journal.pgen.0020070 (PMC1456319; doi:10.1371/journal.pgen.0020070)
Supplement: Supplementary file 4 [file pgen.0020070.sd004.pdf]

Protocol S4- PCR conditions for measuring the number of crossovers and amplifiable genomes.

A. **List of PCR conditions used for detecting recombinants.** The following is a list of the PCR conditions used to preferentially amplify recombinants in each interval. Every PCR reaction contained 0.4 uM of each of the appropriate forward and reverse allele-specific primers, which had 2-4 phosphorothioate bonds and sometimes mismatches at the 3' end. Primers used in the 1st and 2nd round are labeled 1st PCR or 2nd PCR Forward/Reverse (Fwd/Rev), respectively. Red letters in bold refer to mismatches in the primer sequence to improve the difference between the amplification of recombinants over non-recombinants and asterisks denote phophorothioate bonds. These bonds increase the specificity of the primer and also protect the 3' end of the primer from the digestion of the 3'-5' exonuclease activity of the polymerase. Underlined nucleotides in lower case are G/C tails added to increase the melting temperature of the primer. The 1st round of PCR was set up in a separate biosafety cabinet irradiated beforehand with UV light and carried out in a DNA Engine Tetrad 2 (MJResearch). A 0.5ul aliquot of the first round amplification product was added to the 2nd round and amplified using a 7900HT Real-Time PCR System (Applied Biosystems). Both 1st and 2nd round PCR reactions were performed in 384-well plates in volumes of 10 ul. Shown in red before each primer list are the haplotypes of the measured recombinant. The nucleotides in parenthesis represent InDels.

Interval 1:

We only used for this interval a single round of PCR to count the recombinants. Both primers annealed to InDels making the reaction very specific and eliminating the need of a second PCR round with primers annealing to an internal set of SNPs.

Recombinant haplotype: (tt) (tgc)

Primers	SNP	Sequence
1st PCR Fwd	20686 (Marshfield InDel)	gggcCCCAAACCCCAAATAAAAAACT*C*T*T*T
1st PCR Rev	209377 (Marshfield InDel)	GCCTTCCAAGATGGGC*A*G*C*A

Reaction conditions: See methods (Expand Long Template buffer 2)
Cycling conditions:
1st PCR:

92°C 2'

92°C 10''
62°C 30''
68°C 6'
} Repeat 36x

Interval 2:
Recombinant haplotype: (tgc)t ag

Primers	SNP	Sequence
1st PCR Fwd	209377 (Marshfield InDel)	agtaaatggtatagttcc*t*g*c*t
1st PCR Rev	rs461019	CCTATTGAAATAGAAAAAA*T***T***G*C
2nd PCR Fwd	rs462782	cttcagaacatctgtcttttca*a*c*t
2nd PCR Rev	rs461466	TTTGGTCAAACTTACAAACTCA*T*G*A*T

Reaction conditions: See methods (Expand Long Template buffer 2)

Cycling conditions:
1st PCR:

92°C 2'

92°C 10''
52°C 30''
68°C 4'
} Repeat 10x

92°C 15''
52°C 30''
68°C 4'+20''
} Repeat 15x

2nd PCR:

92°C 2'

92°C 10''
63°C 30''
68°C 4'
} Repeat 5x

92°C 15''
63°C 30''
68°C 4'+20''
} Repeat 35x

Interval 3:
Recombinant haplotype: ct tc

Primers	SNP	Sequence
1st PCR Fwd	rs926080	GATCAAGCAGCATTTTCTAGT*G***T***A*C
1st PCR Rev	rs2837238	cggTCTATTTCAACCAATATCCAT*T*G*C*A
2nd PCR Fwd	rs2837237	TCATTCTGGCTTCTGTTCCCA*T***T***A*T
2nd PCR Rev	rs459814	TGGTTTTGCAGGCCTGATCTT*C***C***C*A

Reaction conditions: See methods (Expand Long Template buffer 2)

Cycling conditions:
1st PCR:

92°C 2'

92°C 10''
58°C 30''
68°C 8'
Repeat 25x

2nd PCR:

92°C 2'

92°C 10''
61°C 30''
68°C 7'
Repeat 38x

Interval 4:
Recombinant haplotype: tt ag

Primers	SNP	Sequence
1st PCR Fwd	rs459814	TATCAGGTCATGATCCAG*A***T***A*T
1st PCR Rev	rs2837241	cggTCTATCACAGTTTATAATTTTC*T*C
2nd PCR Fwd	rs2837238	gggcGTTCACTATTACTATTAAAAT*A*A*C*T
2nd PCR Rev	rs2837240	TTTTCTCCCTTGCCT*A*G*G*T

Reaction conditions: See methods (Expand Long Template buffer 2)

Cycling conditions:
1st PCR:

92°C 2'

92°C 10''
54°C 30''
68°C 4'
Repeat 10x

92°C 15''
54°C 30''
68°C 4'+10''
Repeat 15x

2nd PCR:

92°C 2'

92°C 10''
52°C 30''
68°C 4'
Repeat 5x

92°C 15''
52°C 30''
68°C 4'+10''
Repeat 35x

Interval 5:
Recombinant haplotype: tc tc

Primers	SNP	Sequence
1st PCR Fwd	rs741866	CAATTTTGGCTGTCAAGG*G*T*T
1st PCR Rev	rs409989	CCAGTGAAAAGGAATGCTTT*C*T*C*G
2nd PCR Fwd	rs1004663	GTCCACAATGGCTTACCG*A*A*A*C
2nd PCR Rev	rs2837246	CTAGAAAATGAAAGTAAGCC*T*C*A*A

Reaction conditions: See methods (Expand Long Template buffer 2)

Cycling conditions:
1st PCR:

92°C 2'

92°C 10''
62°C 30'' } Repeat 10x
68°C 8'

92°C 15''
62°C 30'' } Repeat 13x
68°C 8'+20''

2nd PCR:

92°C 2'

92°C 10''
52°C 30'' } Repeat 10x
68°C 4'

92°C 15''
52°C 30'' } Repeat 35x
68°C 4'+10''

Interval 6:
Recombinant haplotype: tc at

Primers	SNP	Sequence
1st PCR Fwd	rs2837246	ATGAGTCTTTAAGGAAAA*C*A*A*T
1st PCR Rev	rs398802	AGAGACAAGTTACAGAAAAG*A*T*A
2nd PCR Fwd	rs409989	AAGAACATATTTCATTAATTT*A*C***T***C
2nd PCR Rev	rs2837255	CACTTCATCCCATCA*T*G*A*T

Reaction conditions: See methods (Expand Long Template buffer 3)

Cycling conditions:
1st PCR:

92°C 2'

92°C 10''
56°C 30''
68°C 8'
Repeat 10x

92°C 15''
54°C 30''
68°C 8'+10''
Repeat 18x

2nd PCR:

92°C 2'

92°C 10''
54°C 30''
68°C 5'
Repeat 10x

92°C 15''
52°C 30''
68°C 5'+10''
Repeat 35x

Interval 7:
Recombinant haplotype: a(ag) tt

Primers	SNP	Sequence
1st PCR Fwd	rs2837255	AATGGGGCTAATTATGC*C*C*A*A
1st PCR Rev	rs43859	TTAAATTATTCCCTATGTATGG*A*T*G*A
2nd PCR Fwd	222230<br>Marshfield InDel	CACACAAAGATGACA*G*A*G*G
2nd PCR Rev	rs389885	actaattttgcaagactca*a*t*a*a

Cycling conditions: Expand Long Template buffer 3
1st PCR:

92°C 2'

92°C 10''
54°C 30'' Repeat 10x
68°C 10'

92°C 15''
54°C 30'' Repeat 18x
68°C 10'+10''

2nd PCR: Expand Long Template buffer 2

92°C 2'

92°C 10''
56°C 30'' Repeat 10x
68°C 4'

92°C 15''
56°C 30'' Repeat 35x
68°C 4'+10''

Interval 8:

Recombinant haplotype: gt cg

Primers	SNP	Sequence
1st PCR Fwd	rs398802	GATTTGCTTACTCACAATC*T*A*A*G
1st PCR Rev	rs415573	GGATCTCTGTCTTGTC*A*C***T***T
2nd PCR Fwd	rs389885	gcagctcccttcttt*t*t*g*t
2nd PCR Rev	rs438591	TTAAATTATTCCCTATGTATGG*A*T*G*G

Cycling conditions:

1st PCR: Expand Long Template buffer 3

92°C 2'

92°C 10''
50°C 30''
68°C 8'
} Repeat 10x

92°C 15''
50°C 30''
68°C 8'+10''
} Repeat 18x

2nd PCR: Expand Long Template buffer 2

92°C 2'

92°C 10''
52°C 30''
68°C 6'
} Repeat 10x

92°C 15''
52°C 30''
68°C 6'+10''
} Repeat 35x

Interval 9:
Recombinant haplotype: ta tg

Primers	SNP	Sequence
1st PCR Fwd	rs438591	cggTGAGTTATAAAATGACC*T*T*T*T
1st PCR Rev	rs2299745	AGAAAAGTGGGATGT*G*C*C*C
2nd PCR Fwd	rs415573	CGTAGCACTTGGAATTAGAA*G*A
2nd PCR Rev	rs2299743	aggttcaaagaaggtga*c*a*g*a

Reaction conditions: See methods (Expand Long Template buffer 2)

Cycling conditions:
1st PCR:

92°C 2'

92°C 10''
58°C 30''
68°C 8'
} Repeat 25x

2nd PCR:

92°C 2'

92°C 10''
58°C 30''
68°C 8'
} Repeat 37x

Interval 10:

Recombinant haplotype: tg gg

Primers	SNP	Sequence
1st PCR Fwd	rs2299743	agtgtcaggctgtctct*g*c*a*t
1st PCR Rev	rs2183577	AACCAACCAATGTGTGCAC*T***C***T*C
2nd PCR Fwd	rs2299745	TGGATAAAAGCACAC*A*A*G*G
2nd PCR Rev	rs8127562	cttaaccaatcctc*c*c*a*t

Cycling conditions:

1st PCR: Expand Long Template buffer 3

92°C 2'

92°C 10''
56°C 30'' } Repeat 10x
68°C 8'

92°C 15''
56°C 30'' } Repeat 15x
68°C 8'+10''

2nd PCR: Expand Long Template buffer 3

92°C 2'

92°C 10''
54°C 30'' } Repeat 10x
68°C 10'

92°C 15''
54°C 30'' } Repeat 35x
68°C 10'+10''

Interval 11:

Recombinant haplotype: gg tc

Primers	SNP	Sequence
1st PCR Fwd	rs8127562	actttgggagacc*c*a*g*g
1st PCR Rev	rs2299751	CTGCATATGTCAAAACTCAT*C*G
2nd PCR Fwd	rs2183577	GAGGACAGTCAATTACATT*T*T*T*G
2nd PCR Rev	rs2299749	agcctcagtgtctgttttta*t*t*a*a

Reaction conditions: See methods (Expand Long Template buffer 2)

Cycling conditions:
1st PCR:

92°C 2’

92°C 10’’
56°C 30’’ } Repeat 10x
68°C 5’

92°C 10’’
56°C 30’’ } Repeat 10x
68°C 5’+10’’

2nd PCR:

92°C 2’

92°C 10’’
58°C 30’’ } Repeat 10x
68°C 5’

92°C 10’’
58°C 30’’ } Repeat 35x
68°C 5’+10’’

Interval 12:

Recombinant haplotype: gt cc

Primers	SNP	Sequence
1st PCR Fwd	rs2299749	tgggtatattgttattct*c*a*t*g
1st PCR Rev	rs2091891	tcgtgccacggt*a*c*t*g
2nd PCR Fwd	rs2299751	tctacccctttttagttgtat*g*t*t*t
2nd PCR Rev	rs2299754	ATGGTTGGACTG*G*G*C*G

Reaction conditions: See methods (Expand Long Template buffer 2)

Cycling conditions:
1st PCR:

92°C 2’

92°C 10’’
54°C 30’’ } Repeat 10x
68°C 4’

92°C 15’’
54°C 30’’ } Repeat 15x
68°C 4’+10’’

2nd PCR:

92°C 2’

92°C 10’’
58°C 30’’ } Repeat 5x
68°C 4’

92°C 15’’
58°C 30’’ } Repeat 35x
68°C 4’+10’’

Interval 13 type A:
Recombinant haplotype: cc ca

Primers	SNP	Sequence
1st PCR Fwd	rs2299754	AGTGGTGAGCC*C*C*C*C
1st PCR Rev	rs2276524	ggtcaaatgaacagac*a*g*a*t
2nd PCR Fwd	rs2091891	tgctctgtcacccagg*c*t***t***c
2nd PCR Rev	rs2253861	CATCTTCCTGGATGCTC*A***T***C*G

Reaction conditions: See methods (Expand Long Template buffer 2)

Cycling conditions:
1st PCR:

92°C 2'

92°C 10''
59°C 30'' } Repeat 25x
68°C 6'

2nd PCR:

92°C 2'

92°C 10''
60°C 30'' } Repeat 37x
68°C 6'

Interval 13 type B:
Recombinant haplotype: tg tg

Primers	SNP	Sequence
1st PCR Fwd	rs2299754	AGTGGTGAGCC*C*C*C*T
1st PCR Rev	rs2276524	Ggtcaaatgaacagac*a*g*a*c
2nd PCR Fwd	rs2091891	tgctctgtcacccagg*c*t***t***g
2nd PCR Rev	rs2253861	CATCTTCCTGGATGCTC*A***T***C*A

Reaction conditions: See methods (Expand Long Template buffer 2)

Cycling conditions:
1st PCR:

92°C 2'

92°C 10''
56°C 30'' } Repeat 10x
68°C 4'

92°C 15''
56°C 30'' } Repeat 15x
68°C 4'+10''

2nd PCR:

92°C 2'

92°C 10''
56°C 30'' } Repeat 10x
68°C 4'

92°C 15''
56°C 30'' } Repeat 35x
68°C 4'+10''

Interval 14:
Recombinant haplotype: tg tg

Primers	SNP	Sequence
1st PCR Fwd	rs2253861	TGTCCTTCTTCTAGACTC*C*T*T
1st PCR Rev	rs2837276	ATCTGATTGATGGACAC*T*A*G*C
2nd PCR Fwd	rs2276524	TTAGAGAATGACCTACC*t*t*c*g
2nd PCR Rev	rs2837275	TATGATGATCATTCCTACAA*T*C*T*A

Reaction conditions: See methods (Expand Long Template buffer 2)

Cycling conditions:
1st PCR:

92°C 2'

92°C 10''
56°C 30''
68°C 6'
Repeat 35x

2nd PCR:

92°C 2'

92°C 10''
52°C 30''
68°C 6'
Repeat 37x

Interval 15 type A:
Recombinant haplotype: tg tt

Primers	SNP	Sequence
1st PCR Fwd	rs2837275	CATCTCAACCATGA*C*C*C*T
1st PCR Rev	rs2244287	GCTTCTGAAAAACTG*C*C*T*A
2nd PCR Fwd	rs2837276	GCTTCCAACTAGATGAAATTG*A*T*G
2nd PCR Rev	rs2244189	GGCTAGTTAACTAAACTGA***C***G*A

Reaction conditions: See methods (Expand Long Template buffer 2)

Cycling conditions:
1st PCR:

92°C 2’

92°C	10’’	Repeat 10x
54°C	30’’	
68°C	8’	
92°C	15’’	Repeat 15x
54°C	30’’	
68°C	8’+20’’	

2nd PCR:

92°C 2’

92°C	10’’	Repeat 10x
55°C	30’’	
68°C	8’	
92°C	15’’	Repeat 35x
55°C	30’’	
68°C	8’+20’’	

Interval 15 type B:
Recombinant haplotype: gt cc

Primers	SNP	Sequence
1st PCR Fwd	rs2837275	CCCATCTCAACCATGAC*C*C*G
1st PCR Rev	rs2244287	GCTTCTGAAAAACTG*C*C*T*G
2nd PCR Fwd	rs2837276	GCTTCCAACTAGATGAAATTG*A*T*T
2nd PCR Rev	rs2244189	GGCTAGTTAACTAAACTGA***C***G*G

Reaction conditions: See methods (Expand Long Template buffer 3)

Cycling conditions:
1st PCR:

92°C 2'

92°C 10''
56°C 30''
68°C 8'
} Repeat 10x

92°C 15''
56°C 30''
68°C 8'+10''
} Repeat 15x

2nd PCR:

92°C 2'

92°C 10''
60°C 30''
68°C 6'
} Repeat 10x

92°C 15''
60°C 30''
68°C 6'+10''
} Repeat 35x

Interval 16:
Recombinant haplotype: ca ga

Primers	SNP	Sequence
1st PCR Fwd	rs2244287	ggATAGAATTAATGTGCA*C*A*G*C
1st PCR Rev	rs994810	cccAAGACCAACAATGAA*A*T*A*T
2nd PCR Fwd	rs2244297	TGGGCTGAATTGTCC*T*C*A*A
2nd PCR Rev	rs2299783	CACAACAGGGAGTCATATTT*C*A*C

Reaction conditions: See methods (Expand Long Template buffer 3)

Cycling conditions:
1st PCR:

92°C 2’

92°C 10’’ } Repeat 10x
55°C 30’’
68°C 4’

92°C 15’’ } Repeat 15x
55°C 30’’
68°C 4’+10’’

2nd PCR:

92°C 2’

92°C 10’’ } Repeat 5x
58°C 30’’
68°C 4’

92°C 15’’ } Repeat 35x
58°C 30’’
68°C 4’+10’’

Interval 17:
Recombinant haplotype: ga gt

Primers	SNP	Sequence
1st PCR Fwd	rs2299783	CGGCCATTCACAG*C*T*G*G
1st PCR Rev	rs2299785	ccccATTATAACAATAAATTTATA*C*C*C*A
2nd PCR Fwd	rs994810	cgATTAGAATGTGTCAAGGGA*T*G*A
2nd PCR Rev	rs2299784	TTCCTGCCTGCTG*G*C*T*C

Cycling conditions: Expand Long Template buffer 3
1st PCR:

92°C 2’

92°C 10’’
54°C 30’’
68°C 8’
} Repeat 10x

92°C 15’’
54°C 30’’
68°C 8’+10’’
} Repeat 20x

2nd PCR: Expand Long Template buffer 2

92°C 2’

92°C 10’’
60°C 30’’
68°C 8’
} Repeat 5x

92°C 15’’
60°C 30’’
68°C 8’+10’’
} Repeat 35x

B. List of PCR conditions used to determine the number of amplifiable genomes for each interval.

After extraction of semen and blood DNA, the quantity and purity of the DNA was determined spectrophotometrically. Duplicate aliquots, each consisting of 30ng of genomic blood or sperm DNA, were amplified using a 7900HT Real-Time PCR System (Applied Biosystems) and primers that annealed outside the SNPs used to amplify the recombinants. The reaction conditions used were as follows: 1x Expand Long Template buffer 2 or 3, 0.5mM dNTPs, 0.4uM Outside Fwd primer, 0.4uM Outside Rev primer, 0.1x SYBR Green I (Molecular Probes), and 3.75U of Expand Long Template PCR Enzyme (Roche). The total reaction volume was 50ul. The number of amplifiable genomes was estimated comparing the average of both aliquots to a standard curve consisting of duplicates of 100, 30, 10, and 3 ng of high quality genomic DNA (BD Biosciences). Typically, the number of amplifiable genomes was equivalent to the optical density of the DNA, but for sperm samples the number of amplifiable genomes was often 2-3 lower.

The PCR conditions used to determine the number of amplifiable genomes for each interval are described below.

Interval 1:

Primers	Primers anneal outside SNP:	Sequence
Outside Fwd primer	206866	TGGGAGGTAATTAGGGTTAAGCGAGTT
Outside Rev primer	209377	GAGAGTAGCCCTCCTCCGTTCTGA

Reaction conditions: Expand Long Template buffer 2

Cycling conditions:

92°C 2’

92°C 10’’
62°C 30’’ Repeat 35x
68°C 6’

Interval 2:

Primers	Primers anneal outside SNP:	Sequence
Outside Fwd primer	209377	CTCTGGCACACCTCTCATTGAAGCG
Outside Rev primer	rs2837237	GGACTTGGGGAAAACAATGGGGGAT

Reaction conditions: Expand Long Template buffer 2

Cycling conditions:

92°C 2'

92°C 10''
68°C 10' } Repeat 37x

Interval 3:

Primers	Primers anneal outside SNP:	Sequence
Outside Fwd primer	rs926080	TTACTTCTTCCCACCTGTGCTT
Outside Rev primer	rs2837238	CGGTGTCTCAATGTGCTTATGT

Reaction conditions: Expand Long Template buffer 2

Cycling conditions:

92°C 2'

92°C 10''
58°C 30''
68°C 8' } Repeat 35x

Interval 4:

Primers	Primers anneal outside SNP:	Sequence
Outside Fwd primer	rs2837238	CAACCTGCTGTCCCAGCTAGA
Outside Rev primer	rs2837241	TCCATCTCTTCACTTGTGGATG

Reaction conditions: Expand Long Template buffer 2
Cycling conditions:

92°C 2'

92°C 10''
60°C 30''
68°C 4' } Repeat 35x

Interval 5:

Primers	Primers anneal outside SNP:	Sequence
Outside Fwd primer	rs2837240	AGACTGCAGCTCCAACTATCAA
Outside Rev primer	rs409989	TTCCGGAATAGAGCACAAGTCT

Reaction conditions: Expand Long Template buffer 2

Cycling conditions:

92°C 2'

92°C 10''
60°C 30''   } Repeat 10x
68°C 8'

92°C 10''
60°C 30''   } Repeat 35x
68°C 8'+10''

Interval 6:

Primers	Primers anneal outside SNP:	Sequence
Outside Fwd primer	rs2837246	GCTCCTTAGCTGCCTGTGTTAT
Outside Rev primer	rs2837261	GAAACCATTTGTCTTCCATACTC

Reaction conditions: Expand Long Template buffer 3

Cycling conditions:

92°C 2'

92°C 10''
55°C 30''   } Repeat 40x
68°C 10'

Interval 7:

Primers	Primers anneal outside SNP:	Sequence
Outside Fwd primer	rs2837255	ATGGTAGCCTCACTCTTTCA*T*T*T*C
Outside Rev primer	rs2438591	GACAGGTAAAATCCATTCGTGA

Reaction conditions: Expand Long Template buffer 3

Cycling conditions:

92°C 2’

92°C 10’’
58°C 30’’ } Repeat 40x
68°C 10’

Interval 8:

Primers	Primers anneal outside SNP:	Sequence
Outside Fwd primer	rs398802	CCCCTGATTACCTTACTTCTC
Outside Rev primer	rs415573	GGTAAAATCCATTCGTGATGCT

Reaction conditions: Expand Long Template buffer 3

Cycling conditions:

92°C 2’

92°C 10’’
52°C 30’’ } Repeat 35x
68°C 10’

Interval 9:

Primers	Primers anneal outside SNP:	Sequence
Outside Fwd primer	rs438591	AGGACAGGCTTGGGCATTTA
Outside Rev primer	rs2299745	GAGGCAGAAAAGTGGGATGTG

Reaction conditions: Expand Long Template buffer 2

Cycling conditions:

92°C 2’

92°C 10’’
58°C 30’’ } Repeat 36x
68°C 8’

Interval 10:

Primers	Primers anneal outside SNP:	Sequence
Outside Fwd primer	rs2299742	gcagaagaagagctacaat
Outside Rev primer	rs2183577	TTTACAACCAACCAATGTGTGC

Reaction conditions: Expand Long Template buffer 3

Cycling conditions:

92°C 2’

92°C 10’’
56°C 30’’ } Repeat 40x
68°C 10’

Interval 11:

Primers	Primer anneal outside SNP:	Sequence
Outside Fwd primer	rs8127562	AATGCTTCCCTGTGGAAT
Outside Rev primer	rs2837264	tctcatgaaggacgtaggctct

Reaction conditions: Expand Long Template buffer 2

Cycling conditions:

92°C 2’

92°C 10’’
55°C 30’’ } Repeat 35x
68°C 6’

Interval 12:

Primers	Primers anneal outside SNP:	Sequence
Outside Fwd primer	rs2299751	CCAGCACACTTATTTTTGAGGTT
Outside Rev primer	rs2837270	CAAGTTCCCATTAAAAGACACG

Reaction conditions: 1x Expand Long Template buffer 2

Cycling conditions:

92°C 2’

92°C 10’’
58°C 30’’ } Repeat 35x
68°C 5’

Interval 13:

Primers	Primers anneal outside SNP:	Sequence
Outside Fwd primer	rs2299754	GATGGGAAAGAAAGGGAGGAGT
Outside Rev primer	rs2276524	GGTCAAATGAACAGACAGA

Reaction conditions: 1x Expand Long Template buffer 2

Cycling conditions:

92°C 2’

92°C 10’’
58°C 30’’ } Repeat 35x
68°C 6’

Interval 14:

Primers	Primers anneal outside SNP:	Sequence
Outside Fwd primer	rs2253861	AAGGAAGTCATTGTGAGGGAAA
Outside Rev primer	rs2837276	CCCAGGGAATTTTAACCGATG

Reaction conditions: 1x Expand Long Template buffer 2
Cycling conditions:

92°C 2’

92°C 10’’
58°C 30’’ } Repeat 37x
68°C 6’

Interval 15:

Primers	Primers anneal outside SNP:	Sequence
Outside Fwd primer	rs2837275	CGCTTCAGCTTACAGAGG*A*T*T*T
Outside Rev primer	rs2244297	GAGGGGAGAACACATCTAGCTT

Reaction conditions: 1x Expand Long Template buffer 3

Cycling conditions:

92°C 2’

92°C 10’’
60°C 30’’ } Repeat 45x
68°C 8’

Interval 16:

Primers	Primers anneal outside SNP:	Sequence
Outside Fwd primer	rs2244287	AAGCAGGAGATATTCCAGGTTTC
Outside Rev primer	rs994810	ATTTTCTGGGATCAGGGCAAC

Reaction conditions: 1x Expand Long Template buffer 3

Cycling conditions:

92°C 2’

92°C 10’’
60°C 30’’ } Repeat 10x
68°C 4’

92°C 10’’
60°C 30’’ } Repeat 35x
68°C 4’+20’’

Interval 17:

Primers	Primers anneal outside SNP:	Sequence
Outside Fwd primer	rs2299783	TATCTGGGCTAGAAACGGCCTA
Outside Rev primer	rs2299785	TGTTGGAGAAAAGGTCAATTCC

Reaction conditions: 1x Expand Long Template buffer 2

Cycling conditions:

92°C 2’

92°C 10’’
58°C 30’’
68°C 8’
} Repeat 10x

92°C 10’’
58°C 30’’
68°C 8’+20’’
} Repeat 35x
