# Supplementary material for: High-Resolution Recombination Patterns in a Region of Human Chromosome 21 Measured by Sperm Typing
Source: PLoS Genet. 2006 May 5;2(5):e70. doi: 10.1371/journal.pgen.0020070 (PMC1456319; doi:10.1371/journal.pgen.0020070)

Protocol S5-Comparison of LD and sperm typing in intervals 13 and 15

Figure 1. Comparison of sperm typing measurements averaged for the three individuals and both reciprocal crossovers measured in interval 13 (top) with recombination predicted by the three LD based estimators, LDHat-red, Hotspotter-green, and ABC-red (bottom). Crossover data is found in Supplement 2 and data for LD based estimators is found in Supplement 6.

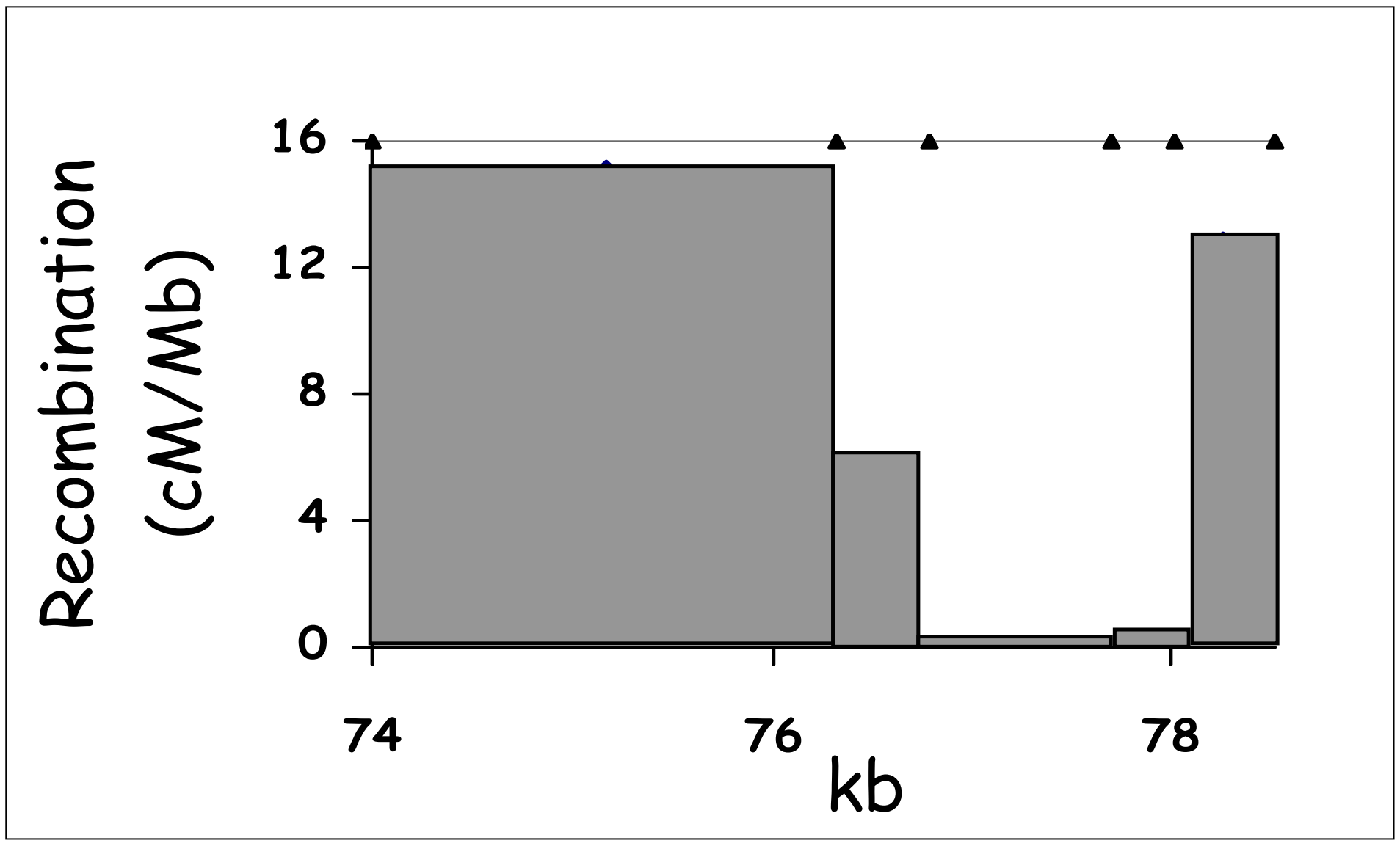

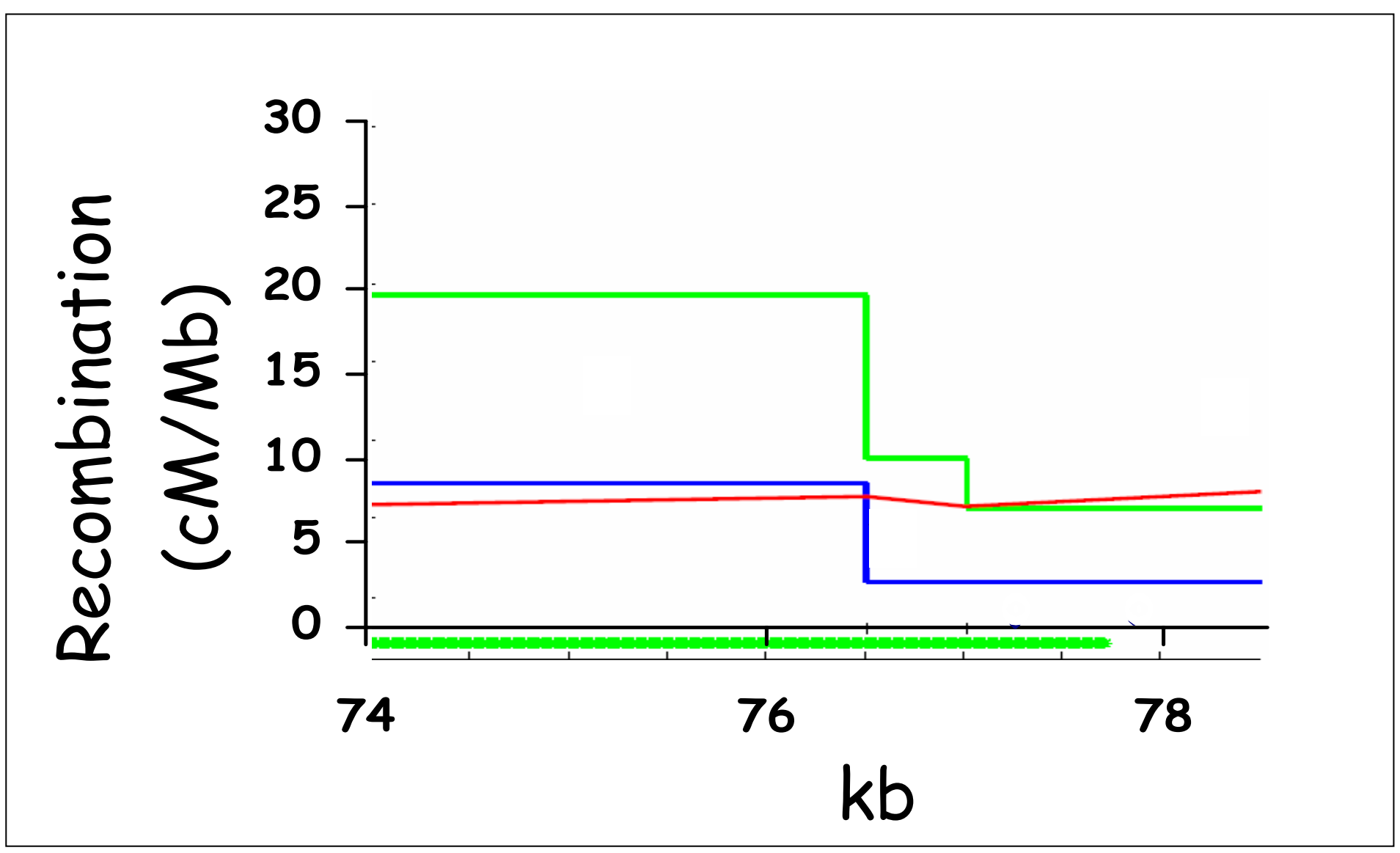

Figure 2. Comparison of sperm typing measurements averaged for the three individuals and both reciprocal crossovers measured in interval 15 (top) with recombination predicted by the three LD based estimators, LDHat-red, Hotspotter-green, and ABC-red (bottom). Crossover data is found in Supplement 2 and data for LD based estimators is found in Supplement 6.

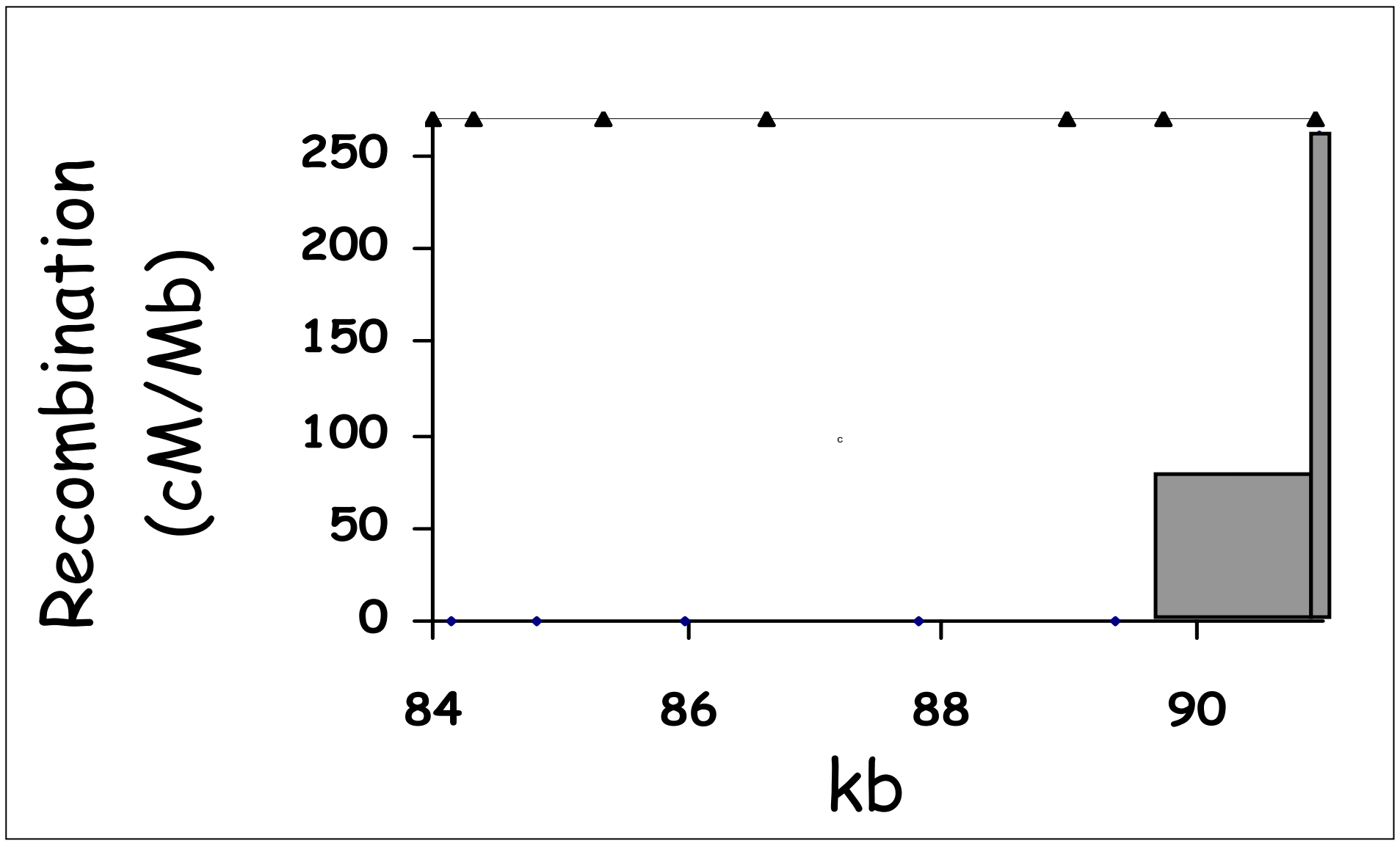

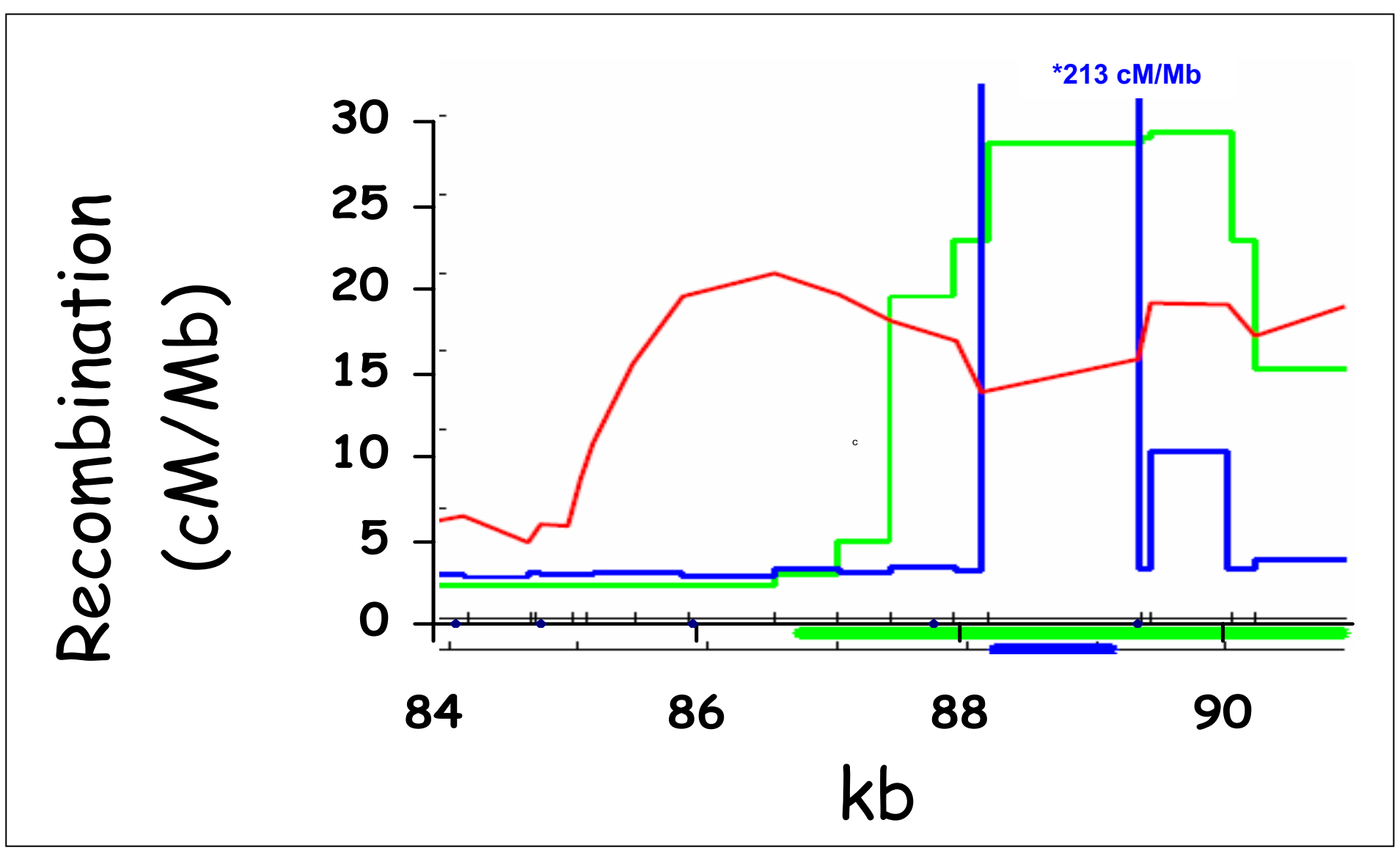

Supplement: Supplementary file 5 [file pgen.0020070.sd005.pdf]
